# Editorial: A Half-Century History of Nutritional Guidance for Pregnant Women in Japan: A Promising Research Target of the DOHaD Study

**DOI:** 10.3389/fendo.2022.942256

**Published:** 2022-07-04

**Authors:** Hiroaki Itoh, Tomoko Aoyama, Yukiko Kohmura-Kobayashi, Takahiro Nemoto

**Affiliations:** ^1^ Department of Obstetrics and Gynecology, Hamamatsu University School of Medicine, Hamamatsu, Japan; ^2^ Liggins Institute, University of Auckland, Auckland, New Zealand; ^3^ National Institutes of Biomedical Innovation, Health and Nutrition, Tokyo, Japan; ^4^ Department of Bioregulatory Science (Physiology), Nippon Medical School, Tokyo, Japan

**Keywords:** developmental origins of health and disease (DOHaD), Japan, low birthweight, weight gain in pregnancy, nutrition

## Introduction

The Research Topic of ‘The Fetal Origins of Metabolic Disorders’ was based on the concept of the Developmental Origins of Health and Disease (DOHaD) theory (1). DOHaD theory connects metabolic disorders of offspring with environmental disruptions during the early critical developmental period. DOHaD theory was devised from the findings of three well-known cohort studies: British (2) and Finnish (3) studies that demonstrated adult health deterioration of low birthweight babies (less than 2,500 g), and a Dutch study (4) of increased risk of adult lifestyle diseases in the people who experienced severe undernourishment *in utero* during the ‘Dutch Famine’ in World War II.

**Figure 1:**
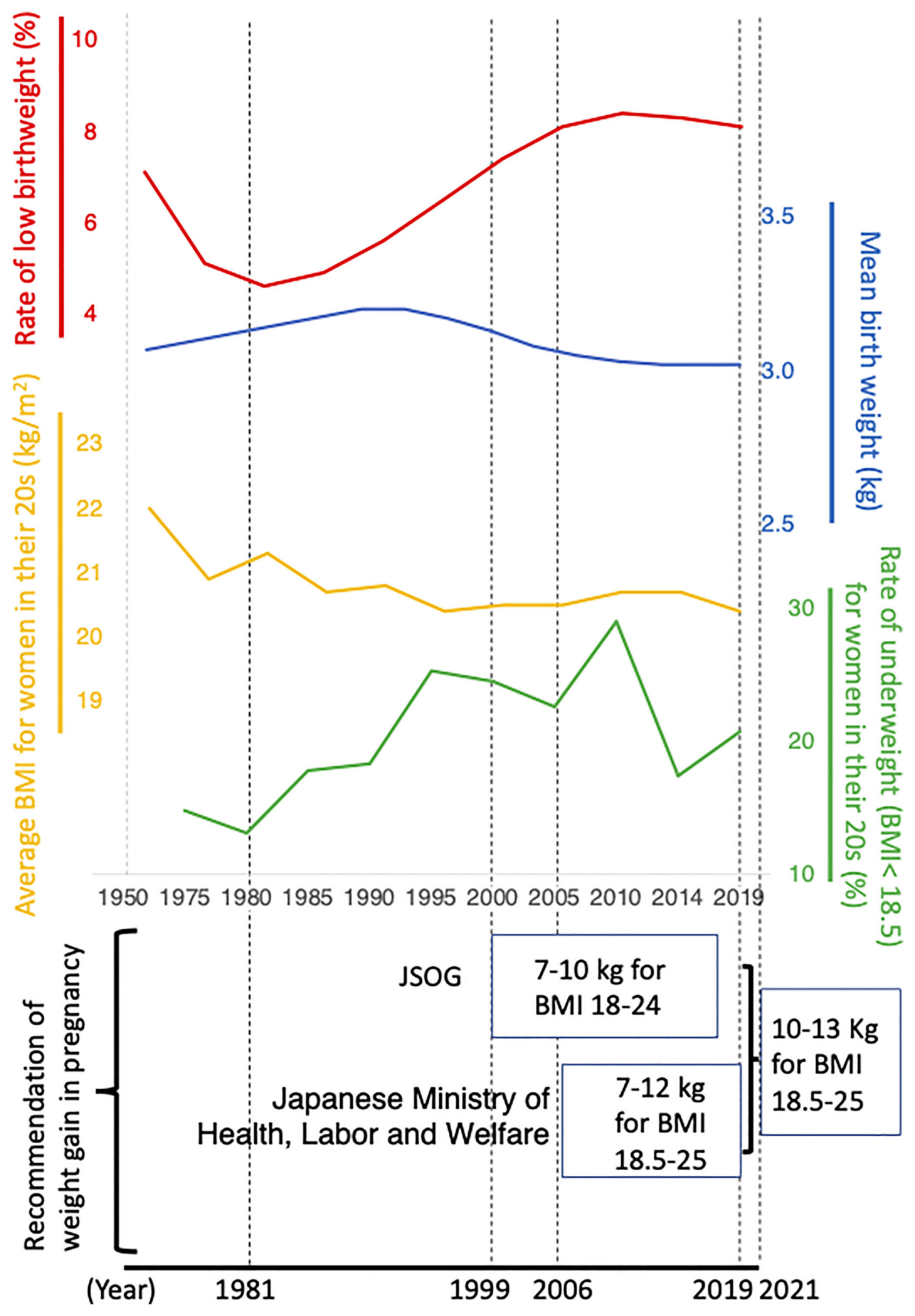
Comparison of the longitudinal changes of rate of low birthweight (%), mean birthweight (kg), average BMI for women in their 20s (kg/m^2^), rate of being underweight (BMI < 18.5) for women in their 20s (%), and recommendation of weight gain in pregnancy at that time in Japan. BMI; body mass index (kg/m^2^). JSOG; Japan Society of Obstetrics and Gynecology. Web data used for **Figure 1** is summarized in **Supplemental Table 1**.

Since the early 1980s in Japan, the rate of low birthweight infants has increased and has remained high at about 10% (**Figure 1**). In this editorial, we introduce the origins and changes of Japanese nutritional guidance for pregnant women and discuss the possible importance of follow-up studies of the offspring to compare health prognoses.

## The History of Nutritional Management of Pregnant Women in Japan Goes Back to Epidemiological Research During World War II

At the end of World War II, there was extreme famine around Amsterdam, the so-called ‘Dutch Famine’. During the famine, pregnant women had low incidence rates of preeclampsia (5) and decreased blood pressure during labour (6). Based on a clinical study (7), the Japan Society of Obstetrics and Gynecology (JSOG) officially recommended energy intake restrictions for pregnant women who developed preeclampsia in 1981 (**Figure 1**) (8). In consideration of the effect of this treatment policy, the Perinatal Committee of JSOG aimed to prevent preeclampsia for general pregnant women and formulated a guideline for maternal weight gain in pregnancy in 1997 that was published in 1999 (**Figure 1**) (9). This guideline recommended weight gain of 7-10 kg during pregnancy for pregnant women with a BMI of 18-24 kg/m^2^ (**Figure 1**) (9).

Independently, the Japanese Ministry of Health, Labour and Welfare published the ‘Optimal weight gain chart during pregnancy’ in 2006, which recommended aiming for a birth weight of 2,500 g to 4,000 g and a weight gain of 7-12 kg during pregnancy for women with a BMI of 18.5 to 25 kg/m^2^ (**Figure 1**) (10). It was a serious problem at that time that there were two different guidelines, JSOG 1999 and Ministry of Health, Labour and Welfare 2006, that were enforced in parallel in Japan (**Figure 1**). Moreover, the purpose of the recommendation was different between the two guidelines (prevention of preeclampsia, and aiming for an appropriate birth weight of 2,500 g to 4,000 g, respectively).

Hytten and Leitch, performed metabolic analysis on pregnant Caucasian women of normal physique and calculated physiological weight gain due to pregnancy to be 12.5 kg (11). It is suggested that the weight gain recommendation of JSOG 1999 as well as Ministry of Health, Labour and Welfare 2006 may be less than the physiological weight gain of pregnant women; however, there is no report that performed an exact metabolic analysis of Japanese pregnant women.

## Withdrawal of 1999 JSOG Recommendations of Weight Gain in Pregnancy

Based on the DOHaD theory, an article by Normile (2018) in ‘Science’ reported that the rate of low birthweight since the early 1980s had increased in Japan and may induce serious health problems in future generations, and that there was considerable involvement of the recommendation of strict weight gain during pregnancy by JSOG 1981 in the continuous decrease of birthweight since the early 1980s in Japan (12). However, JSOG 1981 recommended the restriction of calorie intake only to pregnant women complicated with eclampsia (8), whereas the recommendation of strict weight gain restrictions for general pregnant women for the purpose of preventing preeclampsia was published in 1999 (9) (**Figure 1**), when a decrease in Japanese birthweight had already been occurring for two decades (13). However, this may have had a contribution to this trend.

It is also noted that young Japanese women have a strong desire to be thin (14) and their BMI has continued to decline (**Figure 1**), which may be one of important candidates causing a decrease in Japanese birthweight (13). Nevertheless, in consideration of the ‘Science’ article, the JSOG Perinatal Committee acknowledged that the 1999 JSOG recommendation of weight gain in pregnancy was below the physiological increase and may be harmful for fetuses especially concerning long-term health outcomes, and that there had been little evidence supporting its preventive effect for preeclampsia. Therefore, they decided to officially withdraw the 1999 JSOG recommendation (15).

## 2021 Formulation of New Recommendations of Weight Gain in Pregnancy

From the background described in the previous chapter, a new ‘JSOG reference value for weight gain during pregnancy 2021’ was formulated (16). For pregnant women with a BMI of 18.5 to 25 kg/m^2^, the recommended value for weight gain due to pregnancy was 10-13 kg (16), which was quite a large amount compared to that of JSOG 1999 (7-10 kg for BMI of 18 to 24 kg/m^2^) (9) or Japanese Ministry of Health, Labour and Welfare 2006 (7-12 kg for BMI of 18.5 to 25 kg/m^2^) (10) (**Figure 1**). The Japanese Ministry of Health, Labour and Welfare withdrew the 2006 recommendation and officially accepted that of JSOG 2021 (17).

## Impact of Future Long-Term Cohort Studies in Japan as a Unique Model of DOHaD Population Study

In Japan, mean birthweight decreased and the rate of low birthweight increased from the early 1980s. Rather strict restrictions were recommended between 1991 and 2019 (**Figure 1**); however, this was followed by a sudden increase of the weight gain recommendation in 2021. The average BMI of the childbearing generation has decreased (**Figure 1**). These characteristic changes may indicate distinct differences in intrauterine nutritional conditions between the different generations. Therefore, it is an interesting and valuable research target to investigate the long-term prognosis of Japanese offspring of the different generations and possible associations with maternal dietary conditions.

## Contributions of the Articles Published on the Research Topic

The Research Topic of ‘The Fetal Origins of Metabolic Disorders’ fundamentally consists of: 1) environmental disruption in the early critical period, 2) resultant phenotypic and/or metabolic disorders in the offspring, and 3) programming. As for environmental disruption in the early critical period, Ishiyama et al. reported that there were embryonal nutritional effects, Molle et al. described thrifty eating behavior, Lundy et al. reported vitamin D deficiency, and Nemoto et al. demonstrated the effects of nicotine exposure. Regarding resultant phenotypic and/or metabolic disorders in the offspring, Huang et al. and Ikenoue et al. reported changes of fetal growth or composition, and Suzuki et al. and Zhang et al. reported changes in adipose tissue. Regarding programming, Yang et al., Sato et al., and Liu et al. investigated fibroblast growth factor 19, ghrelin, and fatty acid binding protein 4, respectively; Itoh et al. focused on chronic inflammation, (metaflammation); and Kasuga et al. reported epigenetic changes.

## Author Contributions

All authors contributed to the drafting and editing of this editorial and approved the final version.

## Funding

This work was supported by JSPS KAKENHI Grant Numbers JP20H03823, JP20K09666, and JP20K16886, and AMED under Grant Number JP21gm1310009.

## Conflict of Interest

The authors declare that the research was conducted in the absence of any commercial or financial relationships that could be construed as a potential conflict of interest.

## Publisher’s Note

All claims expressed in this article are solely those of the authors and do not necessarily represent those of their affiliated organizations, or those of the publisher, the editors and the reviewers. Any product that may be evaluated in this article, or claim that may be made by its manufacturer, is not guaranteed or endorsed by the publisher.
