# Supplementary material for: Editorial: A Half-Century History of Nutritional Guidance for Pregnant Women in Japan: A Promising Research Target of the DOHaD Study
Source: Front Endocrinol (Lausanne). 2022 Jul 4;13:942256. doi: 10.3389/fendo.2022.942256 (PMC9289665; doi:10.3389/fendo.2022.942256)
Supplement: Supplementary file 1 [file DataSheet_1.pdf]

Supplemental Table 1: Web data used for drawing Figure 1 (2022 April, confirmed to be web-viewable)

1) National Institute of Health and Nutrition
https://www.nibiohn.go.jp/eiken/kenkounippon21/eiyouchousa/keinen_henka_shintai.html

2) Japanese Ministry of Health, Labour and Welfare
https://www.mhlw.go.jp/toukei/saikin/hw/jinkou/tokusyu/syussyo07/index.html

3) e-Stat: Portal Site of Official Statistics of Japan
https://www.e-stat.go.jp/stat-search/files?page=1&layout=datalist&toukei=00450171&tstat=000001041744&cycle=7&month=0&tclass1=000001139646&cycle_facet=cycle&tclass2val=0
